# Putative SET-domain methyltransferases in *Cryptosporidium parvum* and histone methylation during infection

**DOI:** 10.1080/21505594.2022.2123363

**Published:** 2022-09-15

**Authors:** Manasi Sawant, Sadia Benamrouz-Vanneste, Dionigia Meloni, Nausicaa Gantois, Gaël Even, Karine Guyot, Colette Creusy, Erika Duval, René Wintjens, Jonathan B. Weitzman, Magali Chabe, Eric Viscogliosi, Gabriela Certad

**Affiliations:** a Université de Lille, CNRS, Inserm, CHU Lille, Institut Pasteur de Lille, U1019 – UMR 9017 – CIIL – Centre d’Infection et d’Immunité de Lille, Lille, France; b Unité de Recherche Smart and Sustainable Cities, Faculté de Gestion, Economie et Sciences, Institut Catholique de Lille, Lille, France; c Gènes Diffusion, Douai, France; d PEGASE-Biosciences Plateforme d’Expertises Génomiques Appliquées aux Sciences Expérimentales, Institut Pasteur de Lille, Lille, France; e Service d’Anatomie et de Cytologie Pathologiques, Groupement des Hôpitaux de l’Institut Catholique de Lille (GHICL), Lille, France; f Unit of Microbiology, Bioorganic and Macromolecular Chemistry, Department of Research in Drug Development (RD3), Faculté de Pharmacie, Université Libre de Bruxelles, Brussels, Belgium; g UMR7216 Epigenetics and Cell, Université Paris Cité, Fate, CNRS, Paris, France; h Délégation à la Recherche Clinique et à l’Innovation, Groupement des Hôpitaux de l’Institut Catholique de Lille, Lomme, France

**Keywords:** *Cryptosporidium*, life cycle, histone lysine methyltransferases, epigenetic mechanisms, histone methylation, *C. parvum*-induced colon cancer

## Abstract

*Cryptosporidium parvum* is a leading cause of diarrhoeal illness worldwide being a significant threat to young children and immunocompromised patients, but the pathogenesis caused by this parasite remains poorly understood. *C. parvum* was recently linked with oncogenesis. Notably, the mechanisms of gene expression regulation are unexplored in *Cryptosporidium* and little is known about how the parasite impact host genome regulation. Here, we investigated potential histone lysine methylation, a dynamic epigenetic modification, during the life cycle of the parasite. We identified SET-domain containing proteins, putative lysine methyltransferases (KMTs), in the *C. parvum* genome and classified them phylogenetically into distinct subfamilies (namely CpSET1, CpSET2, CpSET8, CpKMTox and CpAKMT). Our structural analysis further characterized CpSET1, CpSET2 and CpSET8 as histone lysine methyltransferases (HKMTs). The expression of the CpSET genes varies considerably during the parasite life cycle and specific methyl-lysine antibodies showed dynamic changes in parasite histone methylation during development (CpSET1:H3K4; CpSET2:H3K36; CpSET8:H4K20). We investigated the impact of *C. parvum* infection on the host histone lysine methylation. Remarkably, parasite infection led to a considerable decrease in host H3K36me3 and H3K27me3 levels, highlighting the potential of the parasite to exploit the host epigenetic regulation to its advantage. This is the first study to describe epigenetic mechanisms occurring throughout the parasite life cycle and during the host–parasite interaction. A better understanding of histone methylation in both parasite and host genomes may highlight novel infection control strategies.

## Introduction

*Cryptosporidium* belong to the eukaryotic phylum Apicomplexa, which includes parasites that cause malaria and toxoplasmosis [1]. *Cryptosporidium* infection is responsible for diarrhoea in healthy immunocompetent individuals and causes life-threatening disease in immunocompromised individuals [2]. Recent epidemiological studies linked diarrhoeal disease caused by the parasite to early childhood morbidity and mortality in developing countries [3,4]. The environmental resilience nature of the *Cryptosporidium* oocysts allows the parasite to withstand common water treatments such as chlorination [5] and to remain a major cause of waterborne outbreaks in industrialized countries [6,7]. Despite its significant impact on public health, there are currently no vaccine or chemoprophylactic drugs to prevent *Cryptosporidium* infection and very few chemotherapeutic options [8]. The majority of human infections by this protozoan are caused by *Cryptosporidium hominis* and *Cryptosporidium parvum*. Thus, *in silico* analysis of genome sequences of *C. hominis* [9] and *C. parvum* [10] offers new opportunities to uncover drug targets. In parallel, *C. parvum* transcriptomes at the oocyst, excysted sporozoites and intracellular stages, have been widely investigated to understand *Cryptosporidium* life cycle development [11,12], despite the challenges posed by the asynchronous parasite life-cycle. Interestingly, with the availability of the technologies to genetically manipulate the parasite [13], it became possible to analyse the transcriptome at specific intracellular stages *in vitro* as well as *in vivo* [14], leading to the discovery of novel targets for therapeutic interventions.

Strikingly, the compact genome of *Cryptosporidium* compared to other apicomplexan species (e.g. approximately 63 Mb for *T. gondii*), accompanied by the paucity of transcription factors families typically found in eukaryotic organisms [15], suggest major differences in the mechanisms of apicomplexan gene regulation. For instance, *Toxoplasma* has more Apicomplexan Apetala 2 (ApiAP2) transcription factors, one of the major regulatory families in the phylum [16]; however, *Cryptosporidium* appears to rely more on E2F/DP1 transcription factors and gene regulation state of pre-AP dominance [17].

In eukaryotes, epigenetic changes include DNA methylation [18] and histone modifications such as lysine methylation [19] and acetylation [20]. Apicomplexan parasites *Toxoplasma* and *Cryptosporidium* encode putative DNA methyltransferases, but lack detectable DNA cytosine methylation events [21]. On the other hand, novel drugs targeting histone deacetylases (HDAC) and resulting in hyperacetylation have been reported to block parasite differentiation in *Toxoplasma* [22], *Plasmodium* [23] and *Cryptosporidium* [24]. Histone lysine methylation is a sophisticated and dynamic post-translational modification which has been extensively studied in *Toxoplasma* and *Plasmodium* [25]. Lysine methyltransferases (KMTs) shown to methylate Histone 4 lysine 20 (H4K20), regulate cell-cycle progression in these two apicomplexan parasites [26]. Lysine methylation marks in *Plasmodium*, namely H3K4me3 and H3K9me3, participate in regulatory mechanisms of variant surface antigen switching, enabling the parasite to evade the host immune response [27,28]. Novel, parasite-specific methylation events could be promising drug targets; for example, parasite H3K18 methylation was recently described in *Theileria* parasites [29]. Histone methylation is a reversible event that can be removed by histone demethylases. Jumonji-C-terminal (JmjC) domain-containing putative histone demethylases were identified in *T. gondii, P. falciparum, Babesia bovis*, and *T. annulata* [30]. Genome analysis also identified two lysine-specific demethylases (LSD)-like proteins in *T. gondii* [31]. These epigenetic modifications have never been investigated in *Cryptosporidium* parasites.

Intracellular pathogens can also induce alterations of their hosts, employing several strategies to target cellular processes during their complex interactions with host cells [32]. They can evade the barriers imposed by checkpoint responses and can manipulate various defence pathways to increase their survival and transmission. Epigenetic mechanisms could play a fundamental role in the dynamics of host – parasite interactions [32]. Lysine methylation is emerging as a versatile and dynamic post-translational modification that contributes critically to cellular differentiation programs and host–pathogen interactions. One example is the activation of the host methyltransferase SMYD3 to contribute to the transformed host phenotype induced by *Theileria* parasites [33]. Strikingly, epidemiological and experimental studies suggest a potential link between *Cryptosporidium* infection and digestive cancer [34]. However, little is known about the significance of epigenetic variations in *Cryptosporidium* development and in the parasite interactions with its host.

In this study, we aim to characterize the KMTs of *Cryptosporidium* in order to identify lysine methylation events which might be involved in regulating gene expression during the life cycle of the parasite and to evaluate host epigenetic events potentially involved in pathogenicity and parasite-induced transformation.

## Materials and methods

### *In silico* analysis

The protein sequences of the SET domains of several representative KMTs including *Saccharomyces cerevisiae* SET1 (GenBank Accession number EDN62358) and SET2 (NP012367), *Homo sapiens* EZH2 (NP004447), SUV39H1 (BAD96791), SET8 (NP065115), SMYD3 (NP001161212), *Toxoplasma gondii* KMTox (×P002371399) and AKMT (×P 002370918) were retrieved from databases and used as queries to search *C. parvum* homologs by performing BLASTp analysis on the database CryptoDB (http://Cryptodb.org). In parallel, JumonjiC (JmJC)-domain was also used for the search of lysine demethylases (KDMs) in the *C. parvum* genome. The presence of the conserved SET domain within the putative KMTs of *C. parvum* was confirmed by analysing the identified sequences using the InterPro program (http://www.ebi.ac.uk) which integrates the signatures provided from 13 different databases (CATH, CDD, HAMAP, MobiDB Lite, PANTHER, Pfam, PIRSF, PRINTS, PROSITE, SFLD, SMART, SUPERFAMILY and TIGRFAMs). Furthermore, multiple sequence alignment was performed to compare the putative SET domain sequences of *C. parvum* with those of a panel of 31 representative KMTs using the MUltiple Sequence Comparison by Log-Expectation (MUSCLE) software under manual supervision. Simultaneously, a phylogenetic analysis was performed from the same set of SET domain sequences. All positions containing gaps and regions of ambiguous alignment were removed, yielding 116 sites for phylogenetic inference. Full-length alignment and boundaries can be available upon request to the corresponding author. Briefly, phylogenetic trees were constructed using the Neighbour-joining (NJ) and Maximum Likelihood (ML) methods implemented in Mega X [35] using the Jones-Taylor-Thornton (JTT) substitution model. The relative stability of topological elements was assessed using 1000 bootstrap replicates for both NJ and ML.

### Homology modelling

Three-dimensional (3D) models of SET domains identified in the *C. parvum* genome (CpSETs) were built with the automated comparative modelling program Swiss Model Interactive Workspace (https://swissmodel.expasy.org/interactive) using as homologous protein templates, highly resolved X-ray crystal structures of human SET1 (MLL1) Protein Data Bank (PDB) code: 5F6 L; X-ray resolution 1.90 Å, SET2 (SETD2) (6J9J; 1.78 Å), and SET8 (SETD8) (5TEG; 1.30 Å). For each CpSET model developed, the quality of the structure was evaluated by the MolProbity web server [36]. The MolProbity score is a combination of the clash score, rotamer and geometric parameters, and the Ramachandran evaluations into a single score [37]. Lower MolProbity scores are better, meaning good quality structures. The MolProbity server reports also a percentile relative to the score distribution for crystal structures near the resolution of the submitted structure. In case of a modelled structure, the distribution is established covering all resolutions (range of 0Å-99Å). Distance matrix alignment (Dali) server [38] was used to perform pairwise structural alignment between the template and the newly generated CpSET models. The secondary structures were assigned using DSSP algorithm and the ChimeraX software was used to visualize the superimposition of templates and CpSET models [39].

### *Cryptosporidium* oocysts

Oocysts of *C*. *parvum* strain Iowa (purchased from Waterborne™, New Orleans, LA, USA) were stored in phosphate-buffered saline (PBS) with penicillin, streptomycin, gentamycin, amphotericin B and 0.001% Tween 20 at 4°C until use. Absence of bacteria and fungi was assured by testing the oocyst suspensions on both Plate Count Agar and Sabouraud plates at 37°C for 1 week. Oocysts viability was determined as previously described [40].

### In vitro culture

Human ileocaecal adenocarcinoma cells (HCT-8; ATCC CCL-244) were maintained in Dulbecco’s modified eagle medium (DMEM) supplemented with 2 mM L-glutamine, 10% Foetal Bovine Serum (FBS) and antibiotics (100 U/ml penicillin, 100 µg/ml streptomycin). Cells were cultured at 37°C in humidified incubator supplemented with 5% CO_2_. Oocysts excystation was triggered as described previously [41]. After infecting the cells with the parasite, the culture was maintained in RPMI 1640 medium supplemented with 2 mM L-glutamine, 15 mM HEPES buffer, 23 mM sodium bicarbonate, 5 mM glucose, 0.5 µM folic acid, 7 µM 4-aminobenzoic acid, 0.1 µM calcium pantothenate, 50 nM ascorbic acid, 1% (vol/vol) heat-inactivated foetal calf serum, 210 µM gentamycin, 170 µM streptomycin and penicillin (105 U/mL). For negative controls that received no parasites, only maintenance medium was applied onto the monolayers.

### Animal experiment

A total of 10 seven-week-old CB17-SCID mice were obtained from a colony bred at the Pasteur Institute of Lille (France). Mice were administered with 4 mg/L of dexamethasone (Merck, Lyon, France) through drinking water. Infective doses of *C. parvum* (10^5^ oocysts/mouse) were prepared as described previously [42] and inoculated by oral-gastric gavage. In order to quantify parasite shedding, mice faeces were collected and treated as described previously [43]. At 60 days post-infection (PI) or when clinical signs of imminent death appeared, mice were euthanized by carbon dioxide inhalation. Experiments were conducted in the animal facility at the Institute Pasteur of Lille (research accreditation number, D 59,350 009). Animal protocols were approved by the French regional ethical committee with the number APAFIS#9621.

### Histopathology

Ileo-caecal regions were removed from each mouse, fixed in 4% neutral formalin and embedded in paraffin. Sections of 4 µm thick were stained by haematoxylin-eosin-saffron (Leica Autostainer-XL, Rueil-Malmaison, France). Histological sections were analysed using a Leica DMRB microscope equipped with a Leica digital camera connected to an Imaging Research MCID analysis system (MCID software, Cambridge, United Kingdom). Neoplastic lesions at different sites were scored as previously described [42].

### Immunofluorescence assay

Sporozoites were fixed in 4% paraformaldehyde (PAF) for 10 min. After a wash with 1X PBS, the sporozoites were incubated in permeabilization solution (0.2% Triton X-100 in 1X PBS) for 5 min then treated 10 min with blocking solution (0.3 M glycine, 1% BSA, 0.1% Tween 20 in 1X PBS). Finally, the sporozoites were incubated in primary antibody solutions for respective histone lysine methylations (Supplementary Table 1) overnight at 4 °C in a humidified chamber. The primary antibody solution was washed away with 1X PBS and the sporozoites were incubated with secondary antibody solution (Supplementary Table 1) for 1 h at room temperature. Following another wash with PBS, the sporozoites were incubated with the antibody anti-*Cryptosporidium* (Sporoglo, Waterborne™, New Orleans, LA, USA) for 45 min. After a final incubation with DAPI (1 µg/ml) for 15 min, the slides were mounted using Mowiol mounting medium (Mowiol ® 4–88, Sigma, USA). For the *in vitro* staining, HCT-8 cells grown on coverslips in 24 well-plates were infected with 30,000 excysted oocysts per well and fixed at different time points PI: 6 h and 24 h to detect asexual stages and 55 h to detect sexual stages. The staining procedure was similar to that described above for sporozoites. For the *in vivo* staining, ileo-caecal sections of 5 µm thickness were obtained from formalin-fixed and paraffin-embedded specimens and placed on glass slides. The progressive rehydration was followed by an antigen retrieval step using citrate buffer pH 6.5 in a microwave oven for 15 min. After 1 h incubation in blocking buffer (2.5% BSA in 0.1% Tween-201X PBS), the primary antibodies, diluted in blocking buffer, were applied for 1 h at 37°C. After three washes of 5 min with 1X PBS supplemented with 0.1% Tween-20, the slides were incubated in the secondary antibodies for 1 h at 37°C. After a final wash, the slides were counterstained with DAPI and mounted with Mowiol mounting medium. The images were acquired using Zeiss LSM880 confocal microscope and analysed using the ZEN lite Digital Imaging software.

### RNA extraction, cDNA synthesis and real-time quantitative PCR (RT-qPCR)

Total RNA was extracted from infected and non-infected HCT8 cells using NucleoSpin RNA Kit (Macherey-Nagel, Germany). An on-column DNase digestion with a RNase-free DNase was included in the process described by the fabricant to remove any genomic DNA contamination in RNA samples. RNA quality and quantity were determined using Agilent RNA6000 Nano kit by capillary electrophoresis (Agilent 2100 bioanalyzer, Agilent Technologies, Santa Clara, CA, USA). cDNA was synthesized from 1 μg of total RNA using oligo-dT primer and Superscript III reverse transcriptase (RT) in a 20 μl reaction (Invitrogen) according to the manufacturer’s instruction. Each amplification was performed in a volume of 20 µl containing 1 μl of cDNA, 200 nM of each primer and 1X Brilliant III Ultra-Fast SybrGreen qPCR Master Mix (Agilent Technologies). The RT-qPCR reactions were performed on a QIAGEN Rotor-Gene Q instrument (Corbett Research, Qiagen) and included an initial denaturation at 95°C for 3 min followed by a two-step cycling protocol consisting of 45 cycles of denaturation at 95°C during 10 s and annealing/extension at 60°C during 10 s. The PCR cycling program was followed by a standard melt step, stepwise increasing temperature each 5 s by 1°C, ranging from 65°C to 95°C. Primers used for RT-qPCR of putative CpKMTs are listed in supplementary Table 2. The 2^−ΔΔCt^ method was used to calculate the relative expression levels of *KMT g*enes with the constitutively expressed 18S rRNA gene as the internal reference and the ΔC_t_ value of the sporozoite stage as the calibrator.

### Purification of histones and western blot

Parasite histones were enriched by performing a fractionation protocol. Briefly, HCT-8 cells infected and non-infected at different PI time points were incubated in ice cold fractionation buffer (25 mM Tris-HCl pH 8.5, 50 mM NaCl, 0.1% Triton-X100, 1 mM EDTA, 1x protease inhibitor) (cOmplete™ Protease Inhibitor Cocktail, Roche, USA). After dislocating the cells, the lysate was centrifuged at 2,000 g for 10 min at 4°C. Respective cellular fractions (pellet and supernatant) were subjected to histone purification using the EpiQuik^TM^ Total Histones Extraction Kit (Epigentek, OP-0006-100, USA). The purified histone concentration was determined using micro BCA protein assay kit (Pierce, Thermofischer Scientific, USA). Approximately equal amounts of purified histones and parasite histones were separated by 15% sodium dodecyl sulphate polyacrylamide gel electrophoresis (SDS- PAGE) and transferred to nitrocellulose membranes (Millipore, USA). Chemiluminescent detection of bands was carried out by using Super Signal West Femto Maximum Sensitivity Substrate (Thermo Scientific, USA).

### Cloning and protein expression of CpSets

To express the SET domain proteins, putative active domains of CpSET8 (aa residues 402–556) was *de novo* synthetized and cloned into the bacterial expression vector pET15b at the NdeI and BamHI cloning sites which follows *N*-terminal 6x histidine tag sequence (Gencust, Boynes, France). The expression plasmid was amplified in *E. coli* BL21 (DE3) cells. After induction with 0.1 mM isopropyl β-D-1-thiogalactopyranoside (IPTG) at 16°C for 16 h, the cells were collected by centrifugation. The cell pellets were resuspended in lysis buffer (50 mM Tris-HCl, pH 7.5, 150 mM NaCl, 10 mM Imidazole, 1x protease inhibitor, 1 mg/ml lysozyme, 5 µg/ml DNAse). The suspension was sonicated on ice. After centrifugation, the pellet was homogenized in denaturing solubilization buffer (50 mM Na_2_HPO_4_ pH 8.0, 300 mM NaCl, 8 M urea). The supernatant was subjected to Protino® Ni-IDA column (Macherey-Nagel, Germany). The his-tagged CpSET8 was eluted using denaturing elution buffer (50 mM Na_2_HPO_4_ pH 8.0, 300 mM NaCl, 8 M urea, 250 mM imidazole).

### In vitro histone methyltransferase assay

To detect histone methyltransferase activity, 1 µg of recombinant H4 (New England Biolabs) and 100 µM S-adenosyl methionine (SAM) (Sigma, USA) were mixed with recombinant CpSET domains (CpSET8) in methyltransferase buffer (50 mM Tris-HCl (pH 8.5), 5 mM MgCl_2_, 4 mM DTT) at 30°C for 1 h. The reaction was stopped by adding 2x SDS sample buffer, and the reaction mixture was analysed on 15% SDS-PAGE, followed by western blotting with antibodies against tri-methylation of lysine 20 of histone 4 (H4K20me3) at a dilution of 1:1000 (Supplementary table 1).

### Signal quantification

Fluorescence intensity signals between infected and non-infected host tissue were quantified using ImageJ software version 1.52a (NIH, USA). Signal intensity was measured from individual nucleus of infected vs non-infected epithelial cell form intestinal crypts. For the statistical analysis, a mixed model was used to test the relationship between fluorescence intensity markers and group condition taking in account sample repetition. A mixed regression model was created considering fluorescence quantification as the main outcomes and sample identifier as random effect. The general significance level was set at a *p*-value below 0.05. All analyses were performed using packages nlme from the R statistical computing program (Version 4.1.1, date of release 8 October 2021; R Development Core Team, http://www.R—project.org, accessed on 12 January 2022). The data is represented using GraphPad Prism 9.1 (San Diego, California, USA).

## Results

### *In silico* analysis of putative KMTs in *C. parvum* and their SET domains

In order to identify putative KMTs in the *C. parvum* genome, we ran queries to retrieve genes from *C. parvum* containing the SET-domain consensus. We identified 10 putative KMTs with a recognisable SET domain (Table 1).

**Table 1.** List of putative SET-domain KMTs identified in the *C. parvum* genome.

Gene ID	Chromosomal location	Protein size (amino acids)	Domains in addition to SET domain
cgd8_2730	CM000436: 718,307.725208 (+ strand)	2244	PHD, bromo, post-SET
cgd5_400	CM000433: 103,870.106923 (+ strand)	1004	PHD, AWS, post-SET
cgd4_370	CM000432: 90,500.92170 (+ strand)	556	-
cgd4_2090	CM000432: 502,573.504324 (− strand)	583	TPR
cgd5_2340	CM000433: 503,754.505532 (+ strand)	524	-
cgd7_5090	CM000435: 1,177,390.1179244 (− strand)	574	MNYD
cgd1_2170	CM000429: 494,029.495242 (− strand)	366	-
cgd6_1470	CM000434: 350,153.352792 (− strand)	879	AWS
cgd8_3840	CM000434: 894,260.896419 (+ strand)	719	-
cgd6_980	CM000434: 248,189.249592 (− strand)	467	-

^a^PHD – Plant homeodomain.

*AWS – Associated with SET.

*TPR – Tetrapeptide repeat.

**Figure 1.**
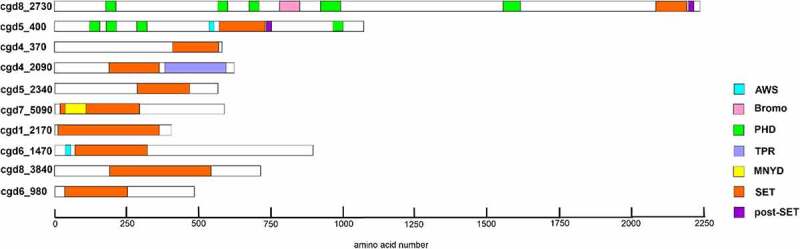
Schematic representation of putative lysine methyltransferases. The image illustrates SET and non-SET domain organizations in 10 putative KMTs of *C. parvum*. The additional domains include, associated with SET (AWS), bromodomain, plant homeodomain (PHD), tetratricopeptide repeat (TPR), Myeloid translocation protein 8, Nervy, and DEAF-1 (MYND). The schematic view of different SET domains vary due to the presence of variable region between motif II and motif III.

The corresponding genes are distributed on six chromosomes and the size of the full-length proteins range from 467 to 2244 amino acids. *In silico* analysis revealed the presence of CpSET proteins orthologues in all *Cryptosporidium* species including *C. muris, C. andersoni, C. hominis, C. meleagridis* and *C. ubiquitum*. Among the known methylated lysines, only H3K79 is known to be methylated by DOT1, a non-SET domain histone methyltransferase in mammals. Unlike SET domain proteins, DOT1 domain containing proteins were observed only in *C. muris* and *C. andersoni*. Analysis of the domain organisation of CpKMTs identified additional domains, including the bromodomain, plant homeodomain (PHD), associated with SET (AWS) domain, tetratricopeptide repeat (TPR) domain and MYND (Myeloid translocation protein 8, Nervy, and DEAF-1) (Table 1) (Figure 1). In contrast, we did not identify any *Cryptosporidium* genes with JmJC-domains which are characteristic of lysine demethylases (KDMs).

**Figure 2.**
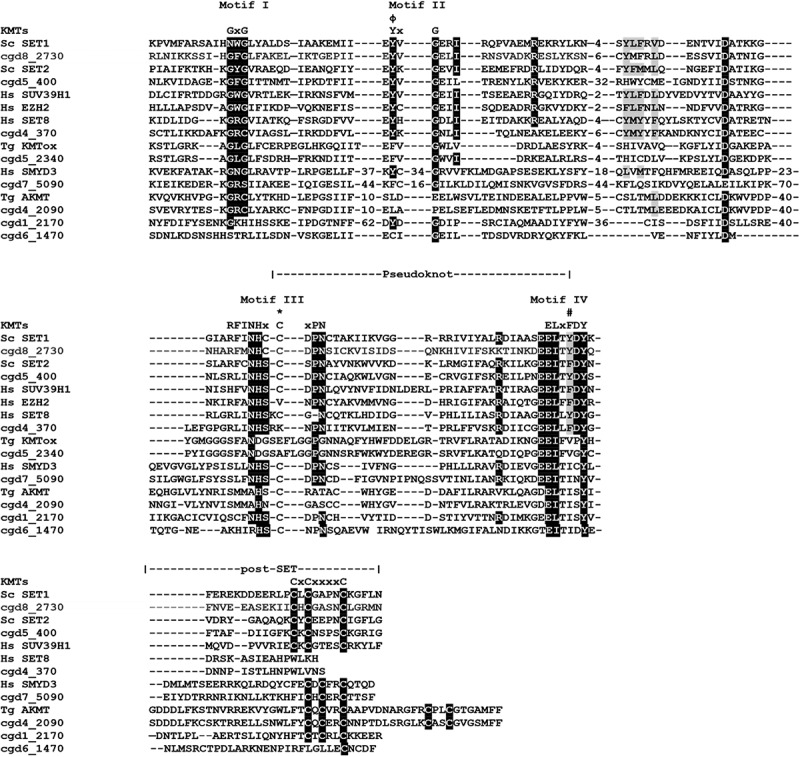
Alignment of SET and post-SET domain sequences of putative CpKmts with representatives of KMT families. The SET domain includes four motifs (I-IV) and their consensus sequences are indicated above the alignment. Motif III and IV are involved in formation of pseudoknot structures to form the active site. The white text on black background indicates identical residues; black text on gray background indicates conserved residues. The residues representing the catalytic site are indicated with phi (Φ). The residues representing F/Y switch are indicated with hash symbol (#). The Cys residue from Motif III that is involved in Zn cluster formation with the post-SET domain are indicated with asterisks (*).

The mammalian SET-domain proteins are classified into seven families including SUV39, SET1, SET2, EZ, RIZ, SMYD and SUV4–20, as well as a few orphan members SET7/9 and SET8 (also called Pr-SET7). We aligned sequences of CpSET domains with representatives of each of these families. Sequence analysis of SET domains of KMTs revealed the presence of four signature motifs; motif I (GxG), motif II (YxG), motif III (RFINHxCxPN) and motif IV (ELxFDY) (Figure 2). Our analysis showed that 8 of the10 predicted CpSET proteins exhibited different levels of similarity to the three catalytically essential motifs GxG, RFINHxCxPN and ELxFDY of the SET domain (Figure 2). This was not the case for the two remaining CpSETs (cgd6_3840 and cgd6_980), which were excluded from further analysis. The motif II is very well conserved in cgd8_2730, cgd5_400 and cgd4_370 compared to other CpSETs. The sequence between motif II and motif III was the most variable region of the SET domain. The canonical preSET domain was not found preceding any of the CpSETs (according to the InterPro database). However, a cysteine (Cys)-rich cluster was observed flanking the *N*-terminal extremity of cgd5_400 and cgd6_1470 SET domains (identified as AWS domain by the InterPro database). Regarding the C-terminal flanking region of the CpSET domain, it is composed of the post-SET domain which contains the CXCX_2-4_C motif, well conserved in cgd8_2730, cgd5_400, cgd1_2170 and cgd6_1470 (also identified as post-domain in InterPro database). All the CpSETs exhibiting the post-SET domain also conserved the Cys residue in the motif III. Indeed, the Cys residues from post-SET domain and motif III together form a channel to accommodate the lysine side-chain. However, cgd7_5090 showed sequence variation in the post-SET motif (CXCX_2_C) similar to SMYD (SET-and MYND-domain containing) and Suv4–20 KMT families. With distinct residues conserved in motif III, cgd5_2340 seems to be part of a separate family of KMT called KMTox. Finally, cgd4_2090 had a conserved post-SET cysteine cluster (CXCX_2_CX_11_CX_2_C) previously described in Apical lysine methyltransferases (AKMT), a cluster of KMTs including only apicomplexan homologues (Figure 2).

**Figure 3.**
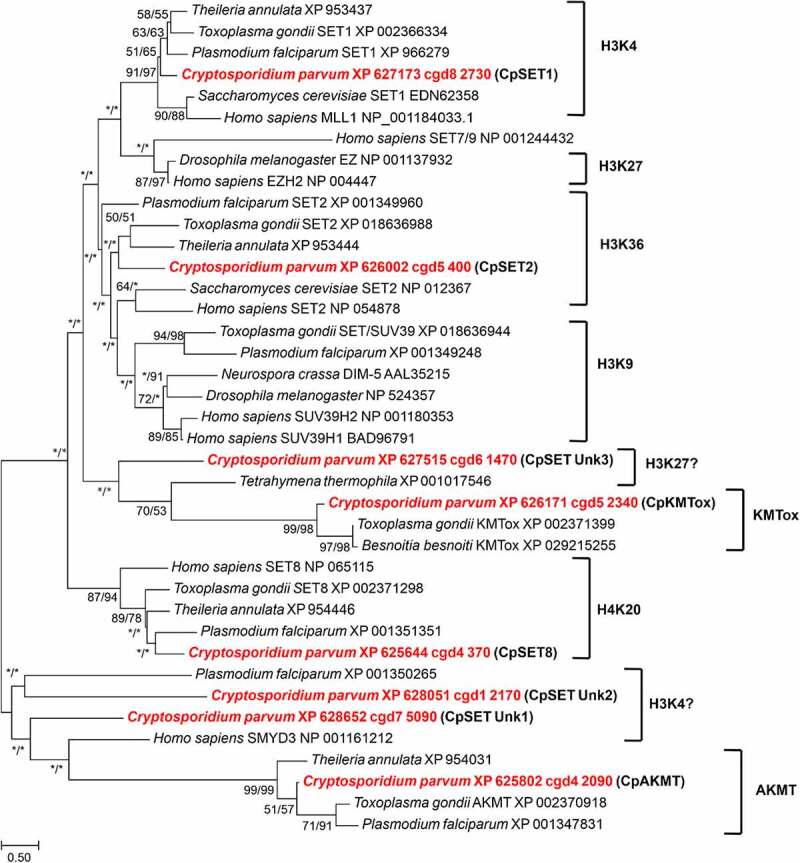
Phylogenetic analysis of SET-domain proteins of *C. parvum*. The putative *Cryptosporidium* sequences are highlighted in red. Numbers near the individual nodes indicate bootstrap values given by NJ (left of the slash) and maximum likelihood (right of the slash). Asterisks indicate nodes with bootstrap values below 50%. Branch lengths are proportional to sequence divergence and can be measured relative to the scale bar. The scale bar indicates the branch length corresponding to 0.50 substitutions per site. The substrate specificities of characterized KMTs clustering with the Cp SET domain proteins are indicated on the right (except KMTox and AKMT which refer to representative KMTs in these groups).

In order to classify the parasite KMTs, we performed phylogenetic analysis of the eight *C. parvum* putative CpSETs, comparing with representatives of different substrate-specific SET domains from yeast, *Drosophila*, humans and Apicomplexa species (Figure 3). We are aware that phylogeny alone is not sufficient to predict function, but the grouping of the CpSETs gives clues for subsequent functional analysis. cgd8_2730 clustered with representatives of the SET1 family of HKMTs such as *S. cerevisiae* SET1 and *H. sapiens* SET1 and was thus assigned as CpSET1. This distribution was strongly supported by bootstrap resampling in NJ (86%) and ML (97%) methods. The clustering of CpSET1 together with previously identified apicomplexan homologues from *T. gondii*, *P. falciparum* and *T. annulata* was also moderately supported by bootstrap values (51% and 65% of the replicates under NJ and ML, respectively). The substrate specificity of members of this family is histone H3K4, so we speculate that parasite H3K4 could be the substrate of CpSET1 (Figure 3) (Table 1). The cgd5_400 gene sequence grouped within the paraphyletic SET2 family of KMTs including enzymes from *S. cerevisiae* and *H. sapiens*, as well as apicomplexan species *T. gondii*, *T. annulata* and *P. falciparum*. Our phylogenetic tree suggested that cgd5_400 belongs to the SET2 KMT family reported to methylate H3K36, justifying the assignment of cgd5_400 as CpSET2 (Figure 3) (Table 1). The cgd4_370 gene is highly homologous to human SET8 and was named CpSET8. This homology was strongly supported by bootstrap values of 87% and 94% according to NJ and ML methods, respectively, suggesting that CpSET8 might methylate the HuSet8 substrate H4K20. The *C. parvum* cgd5_2340 KMT candidate grouped together with KMTox from the apicomplexa *T. gondii* and *Besnoitia besnoiti*, with strong bootstrap values of 99% (NJ method) and 98% (ML method) and was thus assigned as CpKMTox. KMTox is a new family of nuclear KMTs specifically found in Apicomplexa which, similar to AKMTs, also form a distinct apicomplexa-specific clade. The cgd4_2090 *Cryptosporidium* AKMT (CpAKMT) clustered with high bootstrap support (bootstrap values of 99% and 99% according to NJ and ML methods, respectively) with other apicomplexan homologues.

The phylogenetic emergence of the three remaining CpSETs remained uncertain in our analysis (Table 1). We chose to name them CpSET Unk1 (cgd7_5090), CpSET Unk2 (cgd1_2170) and CpSET Unk3 (cgd6_1470) as explained below. Indeed, CpSET Unk3 branched with unsupported bootstrap support at the base of a large group including KMT of the protozoan ciliate *Tetrahymena* and the KMTox of Apicomplexa. Since the KMT of *Tetrahymena thermophila* is known to methylate H3K27, we speculated that CpSET Unk3 could be involved in the H3K27 methylation (Figure 3). The two others CpSET Unk1 and Unk2 are representative of a paraphyletic group that includes the human SMYD3 KMT first described to methylate H3K4 (and subsequently H4K5 and H4K20). The weak grouping of CpSET Unk1 and CpSET Unk2 with SMYD3 implies a possible methylation of H3K4 by these two *Cryptosporidium* KMTs (Figure 3). Finally, unlike other apicomplexan parasites, no *C. parvum* sequences were found to be associated with the SUV39 and EZ families mediating H3K9 and H3K27 methylation, respectively (Figure 3).

### Structural analysis of CpSets

**Figure 4.**
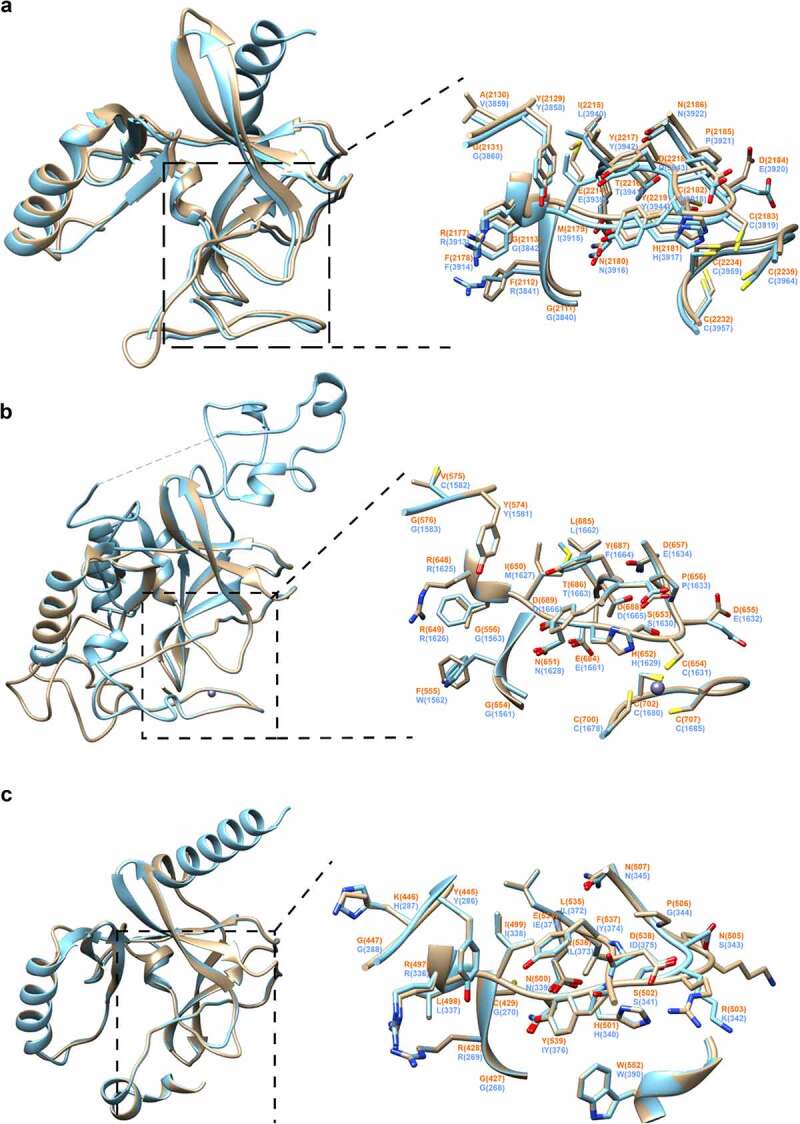
Structural modelling of SET-domain regions of *C. parvum* KMTs. Superimposition of 3D homology models of SET domains (colored in sandy brown) of CpSET1 (a), CpSET2 (b) and CpSET8 (c), superimposed on the crystal structures of SET domains of *Homo sapiens* (HsSET1, HsSET2 and HsSET8, respectively) (colored in cyan). The superimposition of the catalytic site is highlighted in the black dotted box and depicted in an enlarged view with side-chains shown in stick representation (nitrogen and oxygen atoms in blue and red, respectively) and labelled in orange and blue for *C. parvum* and *H. sapiens* structures, respectively.

In order to gain insights into potential functions of the CpSET enzymes, we performed homology modelling of the structures of CpSET1, CpSET2 and CpSET8 using publicly available X-ray crystal structures of homologous enzymes. We found that the overall architecture of the SET domains belonging to different subfamilies of KMTs is nearly identical. The MolProbity scores evaluating the quality of the models were good with values of 1.62 (92^nd^ percentile), 1.73 (88^th^ percentile) and 1.57 (93^rd^ percentile) for CpSET1, CpSET2 and CpSET8, respectively (Supplementary Table 3). Pairwise structure comparisons (DALI program) were used to check whether conserved residues align between CpSETs and the templates. As shown in Supplementary Table 4, CpSET1 shares the highest structural identity with the templates (52%) compared to CpSET2 (43%) and CpSET8 (44%). The visualization of these superimposed structures was performed using ChimeraX software (Figure 4).

Briefly, all the structures contained the specific β-fold identified in KMTs, but not in any other previously characterized AdoMet-dependent methyltransferases. The fold has a series of curved β-strands forming several small sheets that define the core of the SET domain. This β-fold is followed by a knot-like structure which is also observed in all the 3D models of CpSETs. The knot involves a C-terminal β-strand threading through a loop consisting of two β-strands and their connecting region. This represents an archetypal feature of SET domains which consists of residues from the motif II, motif III and post-SET region that enclose the lysine residue and holds it in the appropriate chemical environment and position for methyl transfer by motif I (Figure 4, boxed in black dotted line). The essential residues have similar arrangement in all the SET domains (Figure 4, enlarged images of active site). CpSET1 (Tyr 2129 and Tyr 2217) and CpSET2 (Tyr 574 and Tyr 687) conserved the key tyrosine residues (Figure 4a,b). These Tyr residues are expected to form an intricate network of hydrogen bonds which would place the methyl group in direct line with the N_ε_ of the lysine residue of the histone tail. Structural alignment revealed that in CpSET8 one of these tyrosine residues is replaced by a phenylalanine residue (Tyr 445 and Phe 537) (Figure 4c). This represents the F/Y switch which determines the KMT can mono-, di- or tri-methylate the histone tail [44]. The C-terminal flanking region of different SET-domain families is often divergent, but CpSET1 and CpSET2 exhibited a classical post-SET domain. The prominent feature of this domain is a zinc-binding cage formed by three Cys residues from the C-terminal region, whereas the fourth tetrahedral Cys ligand (CpSET1 Cys 2183; CpSET2 Cys 654) is provided by the loop linking motif II and motif III of the SET domain. The narrow channel formed as a result of Cys interactions accommodates the target lysine and brings the Nε in close proximity to the donor at the opposite end of the channel (Figure 4a,b). In CpSET8, the C-flanking domain consists of a helix and the presence of the Trp 552 residue is likely responsible for interactions with the cofactor (Figure 4c).

### Functional analysis of CpSET8, a putative *Cryptosporidium* SET-domain KMT

**Figure 5.**
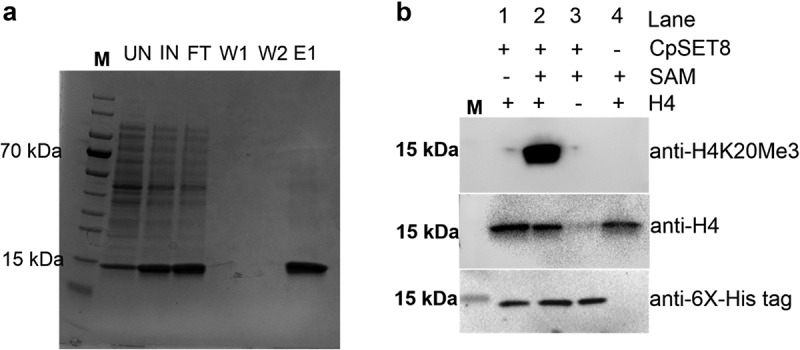
Analysis of recombinant CpSET8 enzymatic activity. (a). Coomassie blue-stained 4–20% precast polyacrylamide gels showing purified CpSET8 showing molecular marker (M), uninduced soluble protein fraction (UN), induced soluble fraction (IN), flow through (FT), washes (W1 and W2), and eluate (E1). (b). Analysis of enzymatic activity showing CpSET8 methylation of H4K20 (anti-H4K20me3 antibody), recombinant human histone H4 (anti-H4 antibody) and recombinant CpSET8 protein (anti-6x-His tag antibody) in the presence of S-adenosyl methionine (SAM).

To investigate whether any of the CpSETs identified in our *in silico* and phylogenetic analysis represent true active KMTs, we produced SET-domain regions of CpSET1 and CpSET8 in a bacterial expression system. Western blot analysis showed detectable amounts of CpSET8 in the soluble fraction after induction and lysis of bacteria (this was not the case for CpSET1) (data not shown). We purified 6x histidine-tagged CpSET8 domain to investigate HKMT activity on recombinant histone H4 (Figure 5a). The recombinant CpSET8 showed strong catalytic activity towards Histone H4, as detected with anti-tri-methylate H4K20 (H4K20me3), antibodies only in the presence of SAM (Figure 5b, lane 2).

### Analysis of parasite KMTs and histone methylation during the *C. parvum* life cycle

**Figure 6.**
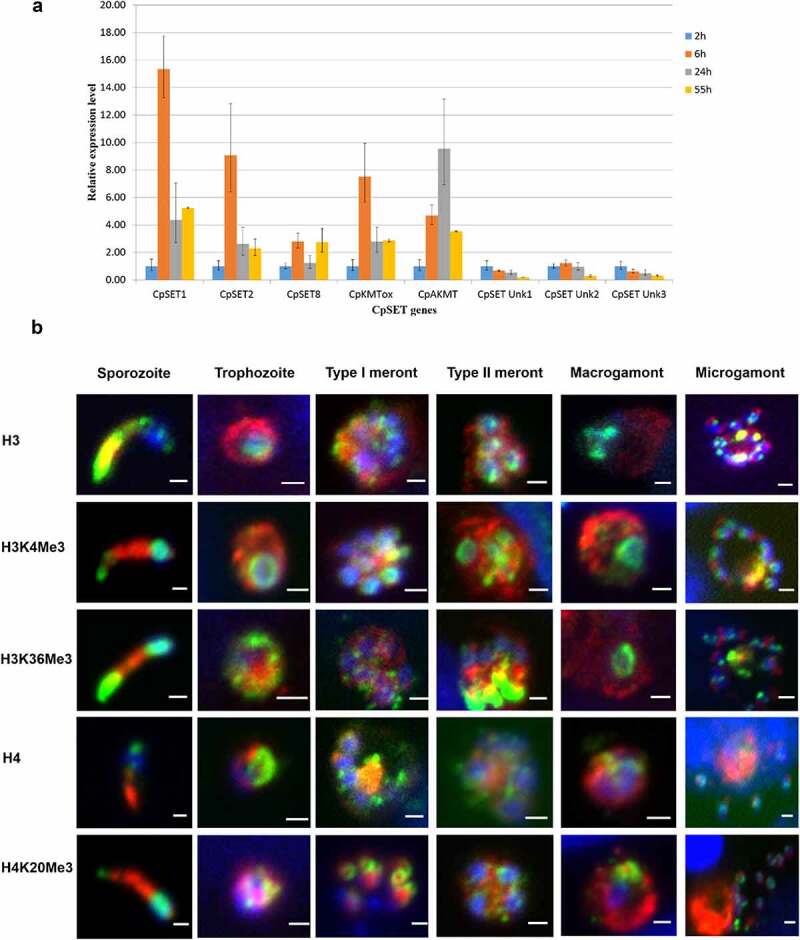
Characterization of KMT gene expression and histone methylation during *C. parvum* infection in HCT-8 *in vitro* culture. (a). RT qPCR analysis illustrating expression of *CpSET* genes during *C. parvum* development *in vitro*. The expression levels were analysed in triplicates and normalized with 18S rRNA gene as the internal control. The ΔC_t_ values at the sporozoite stage were used to calibrate. (b). Immunofluorescence analysis of histone lysine modifications in different stages of *C. parvum*. Co-staining with anti-histone antibodies (H3 and H4) and anti-histone methylation antibodies H3K4me3, H3K36me3 and H4K20me3 (green), anti-*Cryptosporidium* antibody (red) and DAPI (blue).

To determine the expression pattern of CpSET KMTs during the *C. parvum* biological cycle, we performed RT-qPCR analysis of the eight *CpSET* genes using a *in vitro* culture model. We infected human adenocarcinoma cells (HCT-8) with *C. parvum* parasites and monitored the intracellular stage expression of the eight putative *CpSET* genes at different time points (6 h, 24 h and 55 h post-infection, PI) relative to basal expression (2 h post-infection, corresponding to the extracellular sporozoite stage). *CpSET1* showed the highest KMT gene expression, particularly during initial stages of the infection; *CpSET1* exhibited a 14-fold increase at 6 h PI, corresponding to the time-point of predominant trophozoite development. *CpAKMT* expression was 10-fold increased during meront development at 24 h PI. *CpSET2*, *CpSET8*, and *CpKMTox* genes showed relatively high expression during the trophozoite stage (6 h PI), followed by asexual (24 h PI) and sexual stages (55 h PI). When the sexual stages were predominant (at 55 h PI), all the identified putative *CpKMTs* were constitutively expressed. The gene expression of the uncharacterized *CpSET Unk1*, *CpSET Unk2* and *CpSET Unk3* genes was low during intracellular development compared to the extracellular sporozoite stage (Figure 6a).

To further investigate the consequences of the dynamic changes in CpSET gene expression, we analysed histone lysine methylation events during the *C. parvum* life cycle. Importantly, the sequences of the *C. parvum* histone *N*-terminal tails, especially the H3 and H4 lysine residues, are extremely well conserved (Supplementary Figure S1), allowing the use of an array of commercial antibodies recognizing different modified lysine residues. The CpSET1, CpSET2 and CpSET8 putative KMTs are orthologues of SET-domain proteins targeting H3K4, H3K36 and H4K20 methylation (Figure 3). We performed immunofluorescence analysis with antibodies recognizing methylated H3K4, H3K36 and H4K20 lysine residues in cultures of HCT-8 cells infected with *C. parvum* parasites. We observed staining of *C. parvum* chromatin with anti-H3K4me3 antibodies recognizing a broad nuclear distribution through all the developmental stages (Figure 6b). Immunofluorescence analysis with anti-H3K36me3 and anti-H4K20me3 antibodies showed a punctate pattern spread throughout the nucleus, most likely the pericentric heterochromatin, during the intracellular stages (Figure 6b). We quantified the labelling of histone lysine methylation by Western blot analysis (Supplementary Figure S2A). We confirmed that H3K4me3 methylation levels remain consistent during meront development at 24 h PI and microgamont and macrogamont development at 55 h PI. In contrast, H3K36me3 and H4K20me3 methylation levels fluctuated from asexual (24 h PI) to sexual stages 55 h PI (Supplementary Figure S2A). H3K36me3 and H4K20me3 methylation increased by 2–3 fold, when there was a predominance of sexual parasite stages in the culture (Supplementary Figure S2B).

Interestingly, H3 and H4 staining in sporozoites showed an extra-nucleus signal in the apical region. However, it is technically challenging to distinguish between non-nuclear histone localisation and cross-reacting histone mimics. Herein, to overcome with a potential non-specific binding of these antibodies, we verified the specificity by Western blotting and immunofluorescence analysis of sporozoite lysates. Western blotting analysis of *C. parvum* sporozoite lysate detected parasite histones (Supplementary Figure S3), and apical staining in sporozoites was confirmed with both anti-*N*-terminal-H3 and anti-C-terminal-H3 antibodies (Supplementary Figure S4).

### C. parvum infection impacted the methylation of lysine residues in host histones

**Figure 7.**
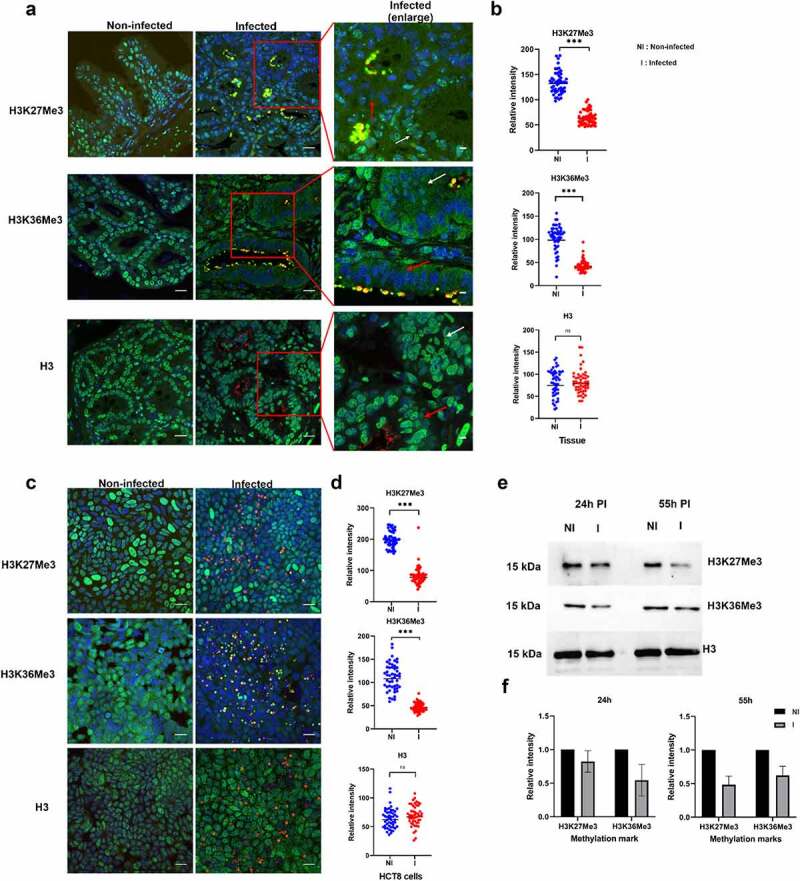
Impact of *C. parvum* infection on host histone methylation events. (a). Immunofluorescence analysis of histone methylation events during *C. parvum* infection *in vivo* at day 60 PI. The red squares represent enlarged images of the infected tissue. Red and white arrows represent infected and uninfected epithelial cells within the intestinal crypt, respectively. Co-staining with antibodies recognizing specific histone methylation (such as H3K36me3, H3K27me3), Histone H3 (green), anti-*Cryptosporidium* antibody (red) and DAPI (blue) of the ileo-caecal region of *C. parvum* infected SCID mice. (b). Quantification of fluorescence intensity signals of anti-methylation antibodies in *C. parvum* infected vs non-infected ileo-cecal tissue. The fluorescence signals were measured for 50 nuclei per sample. For the statistical analysis, a mixed regression model was created considering fluorescence quantification as the main outcome and sample identifier as the random effect. The difference in the fluorescence intensity signals was statistically significant (*p*<0.05). (c). Immunofluorescence analysis of histone methylation events during *C. parvum* infection *in vitro* at 55h PI. (d). Quantification of fluorescence intensity signals of anti-methylation antibodies in *C. parvum* infected vs non-infected HCT8 cells. The fluorescence signals were measured for 50 nuclei per sample. The difference in the fluorescence intensity signals was statistically significant (*p*<0.05). (e). Western blotting analysis of histone methylation events during *C. parvum* infection *in vitro* at 24 h PI (asexual stage), 55 h PI (sexual stages) after purification of histones from host cell. F) the histograms represent relative intensity signals measured in infected HCT-8 cells with respect to non-infected HCT-8 cells. Each sample was normalized to the H3 used as internal control. The graph represents means in triplicate values for Non-infected HCT8 cells (NI), or infected HCT8 cells (I). Scale bar − 20 µm. Results are representative of three independent experiments.

Our model of *C. parvum*-induced colon cancer in SCID mice treated with dexamethasone offers an opportunity to study the impact of infection of host cell methylation *in vivo*. We detected *C. parvum* infection in this animal model, which was confirmed by quantification of oocyst shedding for the entire duration of the experiment. Upon histological examination of the ileo-caecal region of infected animals, the presence of well-differentiated adenocarcinomas invading the submucosae through the *muscularis mucosae* was confirmed after 60 days PI. We used this model to investigate the pathogen-induced modifications of the host phenotype and methylation events by immunofluorescence analysis. As previously described [45], we observed upregulation of the repressive H3K9me3 chromatin mark in the epithelium of the ileo-caecal region of *C. parvum*-infected animals at 60 days PI (Supplementary Figure S5). In contrast, we observed downregulation of the activating methylation mark H3K4me3 (Supplementary Figure S5) in infected hosts. Our most striking finding was the marked deregulation of well-conserved methylation marks associated with transcriptional elongation (H3K36me3) and gene repression (H3K27me3) in the epithelium of the ileo-caecal region of *C. parvum* infected animals at 60 days PI (Figure 7a,b). Notably, these two methylation marks were significantly downregulated in the intestinal crypts where the presence of the parasite was detected (Figure 7a, red arrows). Interestingly, we obtained similar results during *C. parvum* infection of HCT-8 epithelial cells *in vitro*. At 55 h PI, *C. parvum* was observed in all the developmental stages, and the methylation marks were significantly downregulated in infected HCT8 cells (Figure 7c,d). Thus, both *in vivo* and *in vitro* results demonstrate that *C. parvum* infection is associated with downregulation of H3K27me3 and H3K36me3 in the infected intestinal epithelial cells. Further, we performed Western blotting analysis to determine which stages of the parasite could affect the host methylation events during *C. parvum* infection *in vitro*. At 24 h PI, when there is predominant existence of asexual stages of the parasite, H3K36me3 was downregulated by 0.5-fold in infected HCT8 cells, whereas H3K27me3 was downregulated by 0.5-fold in infected cells when sexual forms of the parasite were predominant (Figure 7e,f).

## Discussion

Here, we present the first comprehensive study of *C. parvum* KMTs and histone methylation events during infection, highlighting the potential role of epigenetics in parasite development and pathogenicity. Dozens of SET-domain proteins capable of methylating specific residues have been characterized in higher eukaryotes, driving our search for SET-domain proteins in the *C. parvum* genome (CpSET proteins). The conservation in other *Cryptosporidium* genomes suggests that the parasite ancestor acquired these genes before speciation and divergence within this genus. However, KMTs belonging to the DOT1 family were detected in *C. muris* and *C. andersoni*, but not in *C. parvum*. The absence of DOT1-domain containing lysine methyltransferases in some *Cryptosporidium* species, and in related genera including *Toxoplasma* [46] and *Plasmodium* [30], suggests a secondary loss of the corresponding genes during the evolution of the phylum Apicomplexa. The presence of PHD zinc finger domains [47] and bromodomains [48] suggests that the CpSETs proteins may form protein complexes and interact with chromatin.

Some mammalian KMTs exhibit narrow substrate specificities, often targeting a single lysine within the respective substrates. KMTs also differ in their preference for different methylation states (mono-, di-, or tri-methylation) of lysine residues. In spite of the conserved overall structural plasticity, the variations at the active sites were shown to contribute to their varying substrate specificities [49]. Our analysis of the primary sequence of the SET-domain in CpSET proteins aligned with different families of known KMTs identified the four signature motifs (motif I to IV) involved in transfer of methyl groups in 8 out of the 10 putative CpSETs, suggesting that *C. parvum* KMTs are functionally active. For example, all the residues of the signature motifs of SET and post-SET domains were conserved in the CpSET1 primary amino acid sequence. CpSET1 clustered with SET1 family homologues with high bootstrap support in the present phylogenetic analysis, including the yeast ScSET1 homologue which can methylate H3K4 on gene bodies of actively transcribed genes in *Saccharomyces cerevisiae* [50]. Moreover, the domain organization of CpSET1 includes bromo- and PHD domains which could potentially interact with H3K4me3 [51]. Furthermore, superimposition of CpSET1 3D model with the available crystal structure of human SET1-family MLL1 protein supported the prediction that CpSET1 is a structurally active HKMT. The superimposition analysis shows that CpSET1 conserves all the active site residues (Phe 2159, Tyr 2217, Tyr 2219, Phe 2221, Cys 2156 to Phe 2158) found in the SET-domain of MLL1 [52]. CpSET1 also has conserved key tyrosine residues (Tyr 2129 Tyr 2217) required for the transfer of the methyl group. Superposed structure of MLL1 SET domain with other HKMTs (SET7/9, SET8 and Dim5) revealed that MLL1 has a more spacious active site [52], attributed to a shift in the orientation of SET-I region and C-terminal flanking region in MLL1. This feature is also evident in CpSET1 after superimposition with MLL1. The CpSET1 residues (Cys 2156 to Phe 2158) are conserved which could affect the orientation and free movement of the lysine side chain. Thus, CpSET1 can be predicted to mono-, di- or tri-methylate H3K4. Interestingly, MLL family members (MLL1–4, SET1A and SET1B) are known to methylate H3K4 and have pivotal roles in the regulation of the transcription of genes involved in development, haematopoiesis [52], and cell cycle progression [53]. The H3K4me3 we observed during parasite development could be due to functional CpSET1, representing a MLL1 member of the HKMT family of *Cryptosporidium*, and maintained during development by the relatively high expression of *CpSET1* gene. In addition, H3K4me3 marks the promoter of actively transcribed genes in apicomplexan parasites such as *T. gondii* [54], *T. annulata* [29] and *P. falciparum* [27,52]. Recently, additional components (e.g. RBBP5-ASH2 L) were shown to bind and activate MLL family methyltransferases through a conserved mechanism [55]. An ASH-like histone lysine methyltransferase complex was identified in the genome of *C. hominis* (ChTU502y2012_411g0445, which corresponds to cgd1_740 in *C. parvum*), and the DPY-30 histone methyltransferase complex regulatory subunit was identified in *C. hominis* (Chro. 30,409-t26_1), being cgd3_3620 its orthologous in *C. parvum*. The involvement of other components to maintain HKMT activity will be investigated in further studies.

Analysis of two other *Cryptosporidium* HKMTs (CpSET2 and CpSET8) identified typical post-SET domains and a cysteine-rich *N*-terminal region preceding the SET domain, which both represent features of proteins belonging to the SET2 family of HKMTs [44]. Moreover, the superimposition of 3D homology models of CpSET2 with a template SET-domain of SETD2 (protein data bank: 6J9J.A) revealed 43% identity between the two structures. Strikingly, H3K36me3 methylation is mediated by a single HKMT (SETD2), whereas other H3K36 methyltransferases can only mono- and di-methylate H3K36 [56]. Mutating arginine residue (Arg1625Cys) within the SET-domain resulted in an enzymatically inactive SETD2 failing to tri-methylate H3K36 [57]. The equivalent arginine residue (Arg648) is conserved in CpSET2. Thus, based on the structurally conserved residue, we predict that CpSET2 can tri-methylate H3K36 in *Cryptosporidium*. In addition, the anti-H3K36me3 antibodies detected this methylation mark throughout the parasite life cycle. H3K36me3 is a mark of transcriptional elongation in higher eukaryotes [58] but was linked to repression of *var* genes in *Plasmodium* parasites [59]. Thus, the significance of this methylation mark in *Cryptosporidium* remains to be explored.

The SET8 family of HKMTs were characterized to mono-methylate H4 in humans [60]. The identification and characterization of SET8-related homologs in Apicomplexa, such as *Plasmodium* [30] and *Toxoplasma* [26], showed that this enzyme is not restricted to metazoans [49]. Our phylogenetic analysis suggested that CpSET8 may also methylate H4K20. These findings reinforce the importance of histone methylation in chromatin structure and function in Apicomplexa. The superimposition of 3D homology model of the SET-domain of CpSET8 with a template SET domain from SET8 (protein data bank: 5teg.A) revealed 44% identity between the two structures. The conserved tyrosine residues (Tyr 245 and Tyr 334) within the active site of human SET8 maintain an intricate network of hydrogen bonds to position the side chain of only mono-methylated lysine residues [60]. In contract, Dim-5, a HKMT which can tri-methylate its target lysine, can accommodate mono-, di- and tri-methylated lysine in its active site. This characteristic of Dim-5 was attributed to the replacement of one of the tyrosine residues to phenylalanine residue (Tyr 178 and Phe 281) [61]. Interestingly, structural alignment revealed that in CpSET8 one of these tyrosine residues is replaced by phenylalanine (Tyr 445 and Phe 537). Thus, CpSET8 may be capable of adding multiple methyl group to its target lysine. Based on our structural analysis, we hypothesize that CpSET8 might methylate H4K20me1, H4K20me2 and H4K20me3 like *T. gondii* SET8 [26] in a cell-cycle dependent manner [26]. We showed that recombinant CpSET8 can methylate H4K20 *in vitro* and that both the enzyme and the mark are dynamic during parasite infection. We cannot exclude the possibility that CpSET8 also methylates other targets. However, unlike other HKMTs, such as SMYD3, which are known to have multiple targets [61,62], the SET8 family enzymes appear restricted to a single target, i.e. H4K20 [44]. We propose that CpSET8 can methylate H4K20 and further studies mutating the predicted catalytic residues of the whole protein could reveal the function of the SET domain

CpAKMT was another identified KMT which differed in the C-terminal region, where it retains two extra cysteines in addition to the post-SET domain. Phylogenetic analysis clustered CpAKMT with its homologues from other Apicomplexa, representing a sister-group with HsSMYD3, as described in previous evolutionary studies of *Plasmodium* [30] and *Toxoplasma* KMTs [63]. The lack of a MYND zinc-finger domain, a fundamental feature of SMYDs, classified this group of AKMT homologues as a distinct family. Structural comparison between the AKMT of *T. gondii* and SMYD proteins suggested that features specific to AKMT are necessary for dimerization and specific function outside the nucleus [63]. The *T. gondii* AKMT [64] is localized at the apical complex and associated with parasite motility and egress [65]. Interestingly, our immunofluorescence analysis detected the labelling of lysine methylations at the apical region of *C. parvum* sporozoites and merozoites and the *CpAKMT* gene was expressed during the merozoite development and egress stages. *P. falciparum* histones released from the parasite can exert a disruptive effect on the endothelial barrier function to induce pro-inflammatory responses [66]. Histone modification such as H3K9me1 in *P. falciparum* were linked to the parasitophorous vacuole and host–parasite interactions [67]. Further studies will explore whether CpAKMT is localized at the apical region and can methylate extra-nuclear parasite histones or non-histone proteins to assist in motility. Cytoplasmic SMYD3 may also be a non-histone methyltransferase [68].

The KMTox enzyme in *T. gondii* contains a High Mobility Group (HMG) domain which recognizes bent DNA and allows the SET-domain to methylate histones H4 and H2A *in vitro* [69]. The CpKMTox lacks the HMG domain, but our phylogenetic analysis based on SET-domain sequences clustered together all apicomplexan KMTox members with a high bootstrap value, suggesting that CpKMTox might be an H4/H2A-specific methyltransferase. Although TgKMTox was reported to form a distinct clade with no obvious homologues [69], our phylogenetic analysis identified other apicomplexan parasites retaining KMTox, such as *C. parvum* and *B. besnoiti*, that could represent a new clade of KMTs only found in this group of protozoa.

The nine cysteine residues of the pre-SET domain usually found in the SUV39 family of KMTs [70] were not identified in CpSETs and no CpSETs clustered in our phylogenetic tree with homologues of this family [26]. H3K9 methylation may not be important in *C. parvum* or another enzyme may carry out this function. Several enzymes (CpSET1, CpSET2, CpSET Unk2 and CpSET Unk3) contain conserved cysteine residues in the post-SET region and motif III which could be important for accommodating the target lysine side chain. However, CpSET Unk2 and CpSET Unk3 lacked the motif I signature and could not be grouped together with known KMT families. CpSET Unk1 presented a variant post-SET domain, similar to SMYD or SUV4–20 families, and a MYND zinc finger motif, suggesting that it could be a SMYD homologue targeting H3K4 [61]. It was difficult to characterize these enzymes further and the low expression levels of *CpSET Unk1*, *CpSET Unk 2* and *CpSET Unk 3* suggest that they may not play a role in parasite development. Moreover, these CpSETs could also target non-lysine residues such as histidine, as seen for SETD3, and any of the CpSET proteins could also methylate non-histone targets. Finally, the lack of JmjC-domain proteins in *Cryptosporidium*, raise questions about demethylation dynamics [30,71] or alternative demethylating enzymes.

We also provide new insights into the effect of *C. parvum* infection on the host histone lysine methylation events. We observed modulation of distinct lysine methylation marks in *C. parvum* infected mice and HCT-8 cells, i.e. significant loss H3K36me3 and H3K27me3 methylation upon infection. Polycomb repressive complex 2 (PRC2) is responsible for H3K27 methylation via the EZH2 methyltransferase [72] which is deregulated in multiple cancers [73]. H3K36me3 marks the body of actively transcribing genes and plays a role transcriptional fidelity, mRNA splicing and DNA damage repair and is mutated in some human tumours [74]. It will be interesting to explore whether H3K36me3 and H3K27me3 methylation levels contribute to *C. parvum*-induced neoplasia in our model [75]. This could mirror other oncogenic pathogens that target methylation events [76,77] and signalling pathways (e.g. PI3K/AKT) [78] linked to downregulated methylation in gastric cancers [79], as well as Epithelial Mesenchymal Transition (EMT) events associated with *C. parvum* infection [43]. We hypothesize that *Cryptosporidium* acts in a similar way to *T. gondii* to impair the histone modifications at the promoters of response genes, such as IFN-induced genes [80]. This is consistence with our recent findings that *Cryptosporidium* resists the IFN response and downregulates the expression of anti-microbial peptides such as α-defensins [43]. Nevertheless, host immune response may modulate methylation and some of the histone modifications could be the result of an inflammatory response to infection. Further studies are required to elucidate this aspect and to understand how *Cryptosporidium* hijacks the host epigenetic machinery to escape host immune responses.

In conclusion, our study represents a first step in the characterization of lysine methyltransferases during *C. parvum* infection, opening avenues for anti-parasite drug discovery. Histone and non-histone targets offer an unexplored territory of epigenetic modulations in *C. parvum* infection to deepen our understanding of the dynamics of host–parasite interactions.

## Supplementary Material

Supplemental Material

## Data Availability

The authors confirm that the data supporting the findings of this study are available within the article and the supplementary materials.
