# Supplementary material for: Putative SET-domain methyltransferases in Cryptosporidium parvum and histone methylation during infection
Source: Virulence. 2022 Sep 15;13(1):1632–50. doi: 10.1080/21505594.2022.2123363 (PMC9487757; doi:10.1080/21505594.2022.2123363)
Supplement: Supplemental Material [file KVIR_A_2123363_SM0483.docx]

....|....| ....|....| ....|....| ....|....| ....|....|

10 20 30 40 50

**Hs H3.1**  ARTKQTARKS TGGKAPRKQL ATKAARKSAP ATGGVKKPHR YRPGTVALRE

**Hs H3.2**  ARTKQTARKS TGGKAPRKQL ATKAARKSAP ATGGVKKPHR YRPGTVALRE

**Hs H3.3**  ARTKQTARKS TGGKAPRKQL ATKAARKSAP STGGVKKPHR YRPGTVALRE

**Mm H3.1**  ARTKQTARKS TGGKAPRKQL ATKAARKSAP ATGGVKKPHR YRPGTVALRE

**Mm H3.2**  ARTKQTARKS TGGKAPRKQL ATKAARKSAP ATGGVKKPHR YRPGTVALRE

**Mm H3.3**  ARTKQTARKS TGGKAPRKQL ATKAARKSAP STGGVKKPHR YRPGTVALRE

**Cp H3**  ARTKQTARKS TGGKAPRKQL ASKGARKSAP VTGGVKKPRR YRPGTVALRE

....|....| ....|....| ....|....| ....|....| ....|....|

60 70 80 90 100

**Hs H3.1**  IRRYQKSTEL LIRKLPFQRL VREIAQDFKT DLRFQSSAVM ALQEACEAYL

**Hs H3.2**  IRRYQKSTEL LIRKLPFQRL VREIAQDFKT DLRFQSSAVM ALQEASEAYL

**Hs H3.3**  IRRYQKSTEL LIRKLPFQRL VREIAQDFKT DLRFQSAAIG ALQEASEAYL

**Mm H3.1**  IRRYQKSTEL LIRKLPFQRL VREIAQDFKT DLRFQSSAVM ALQEACEAYL

**Mm H3.2**  IRRYQKSTEL LIRKLPFQRL VREIAQDFKT DLRFQSSAVM ALQEASEAYL

**Mm H3.3**  IRRYQKSTEL LIRKLPFQRL VREIAQDFKT DLRFQSAAIG ALQEASEAYL

**Cp H3**  IRRFQRSTEL LIRKLPFQRL VREIAQDFKT DLRFQSQAVM ALQEAAEAYL

....|....| ....|....| ....|....| ....|.

110 120 130

**Hs H3.1**  VGLFEDTNLC AIHAKRVTIM PKDIQLARRI RGERA.

**Hs H3.2**  VGLFEDTNLC AIHAKRVTIM PKDIQLARRI RGERA.

**Hs H3.3**  VGLFEDTNLC AIHAKRVTIM PKDIQLARRI RGERA.

**Mm H3.1**  VGLFEDTNLC AIHAKRVTIM PKDIQLARRI RGERA.

**Mm H3.2**  VGLFEDTNLC AIHAKRVTIM PKDIQLARRI RGERA.

**Mm H3.3**  VGLFEDTNLC AIHAKRVTIM PKDIQLARRI RGERA.

**Cp H3**  VGLFEDTNLC AIHAHRVTIM PKDIQLARRI RGER..

....|....| ....|....| ....|....| ....|....| ....|....|

10 20 30 40 50

**Hs H4**  SGRGKGGKGL GKGGAKRHRK VLRDNIQGIT KPAIRRLARR GGVKRISGLI

**Mm H4**  SGRGKGGKGL GKGGAKRHRK VLRDNIQGIT KPAIRRLARR GGVKRISGLI

**Cp H4**  SGRGKGGKGL GKGGAKRHRK VLRDNIQGIT KPAIRRLARR GGVKRISALI

....|....| ....|....| ....|....| ....|....| ....|....|

60 70 80 90 100

**Hs H4**  YEETRGVLKV FLENVIRDAV TYTEHAKRKT VTAMDVVYAL KRQGRTLYGF

**Mm H4**  YEETRGVLKV FLENVIRDAV TYTEHAKRKT VTAMDVVYAL KRQGRTLYGF

**Cp H4**  YEEVRGVLKA FLETVIKDAV TYTEYARRKT VTAMDVVHAL KRQGKTLYGF

....|....|

110

**Hs H4**  GG........

**Mm H4**  GG........

**Cp H4**  GG........

**Supplementary Figure 1.** Multiple sequence alignment of histone proteins from *Homo sapiens*, *Mus musculus* and *C. parvum.* Highly conserved lysine residues (K) are highlighted in red. The uniport accession numbers of the sequences used are Hs H3.1 (P68431), Hs H3.2 (Q71DI3), Hs H3.3 (P84243), Hs H4 (Q5CV68), Ms H3.1 (P84228), Ms H3.2 (P84244), Ms H3.3 (P68433), Ms H4 (P62806), Cp H3 (Q5CUJ9) and Cp H4 (Q5CV68).


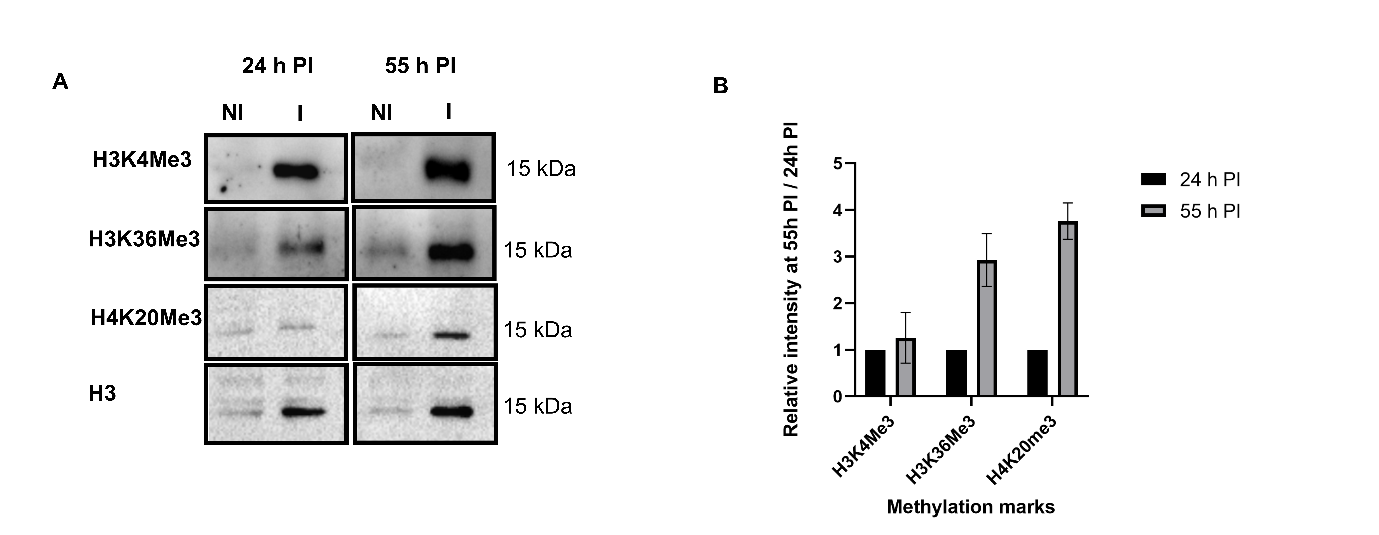


**Supplementary Figure 2**. Western blot analysis of parasite histone methylation marks. (A). Western blotting analysis of histone lysine methylation modifications during *C. parvum* development *in vitro* at 24 h (asexual stages) and 55 h (sexual stages) PI after purification of histones from the parasites. (B). The histograms represent the relative intensity signals of methylation marks in the parasite at 55h PI relative to 24h PI. Each sample was normalized to the H3 used as internal control. The graph represents means in triplicate values. NI – Non-infected HCT-8 cells. I – Infected HCT-8 cells. Scale bar – 1 µm. Results are representative of three independent experiments.


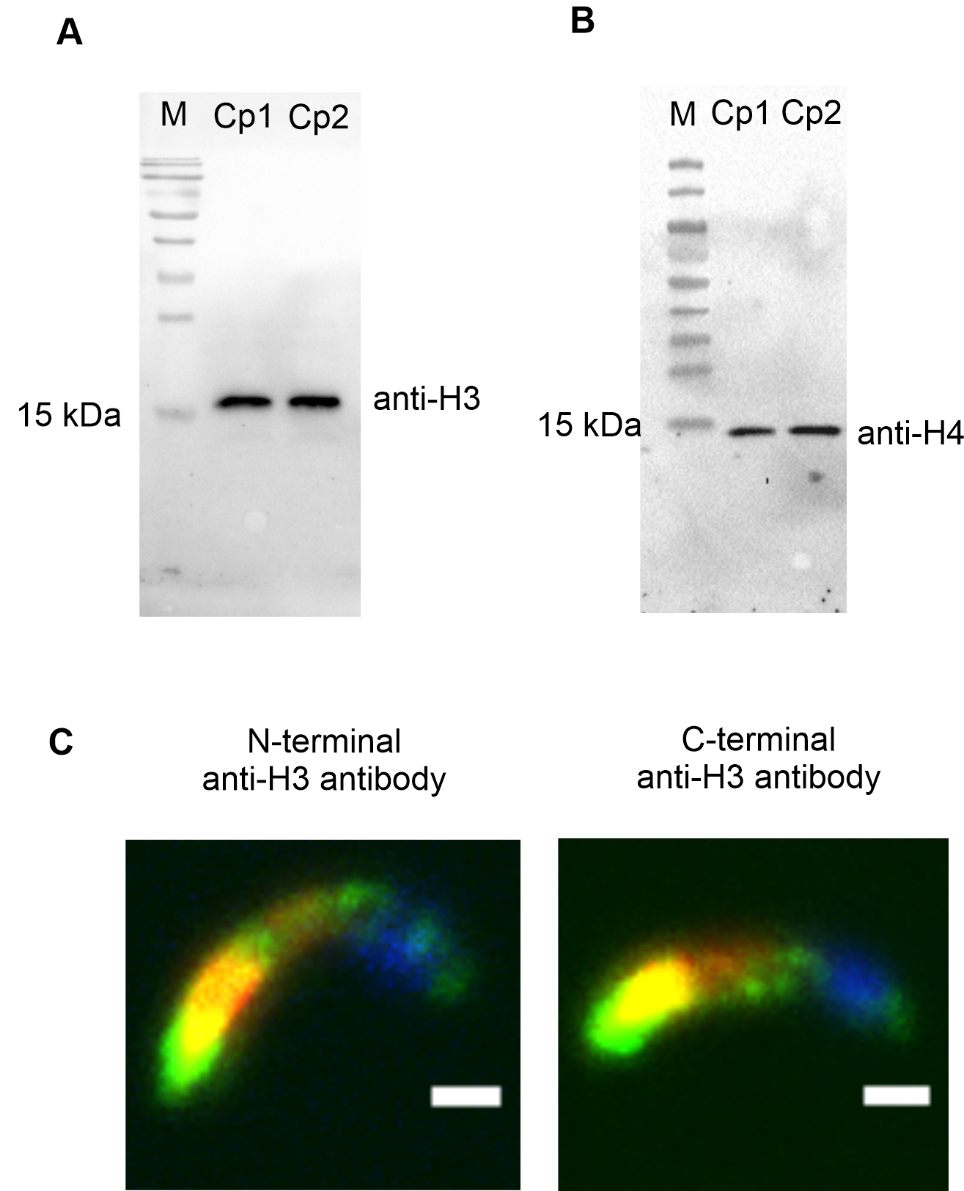


**Supplementary Figure 3**. Western blotting analysis of *C. parvum* sporozoite lysate to detect parasite histones. Chemiluminescent signals detected histone 3 using a commercially available anti-H3 antibody (Ab18521). Cp1 and Cp2 represent duplicates. M, molecular ladder.


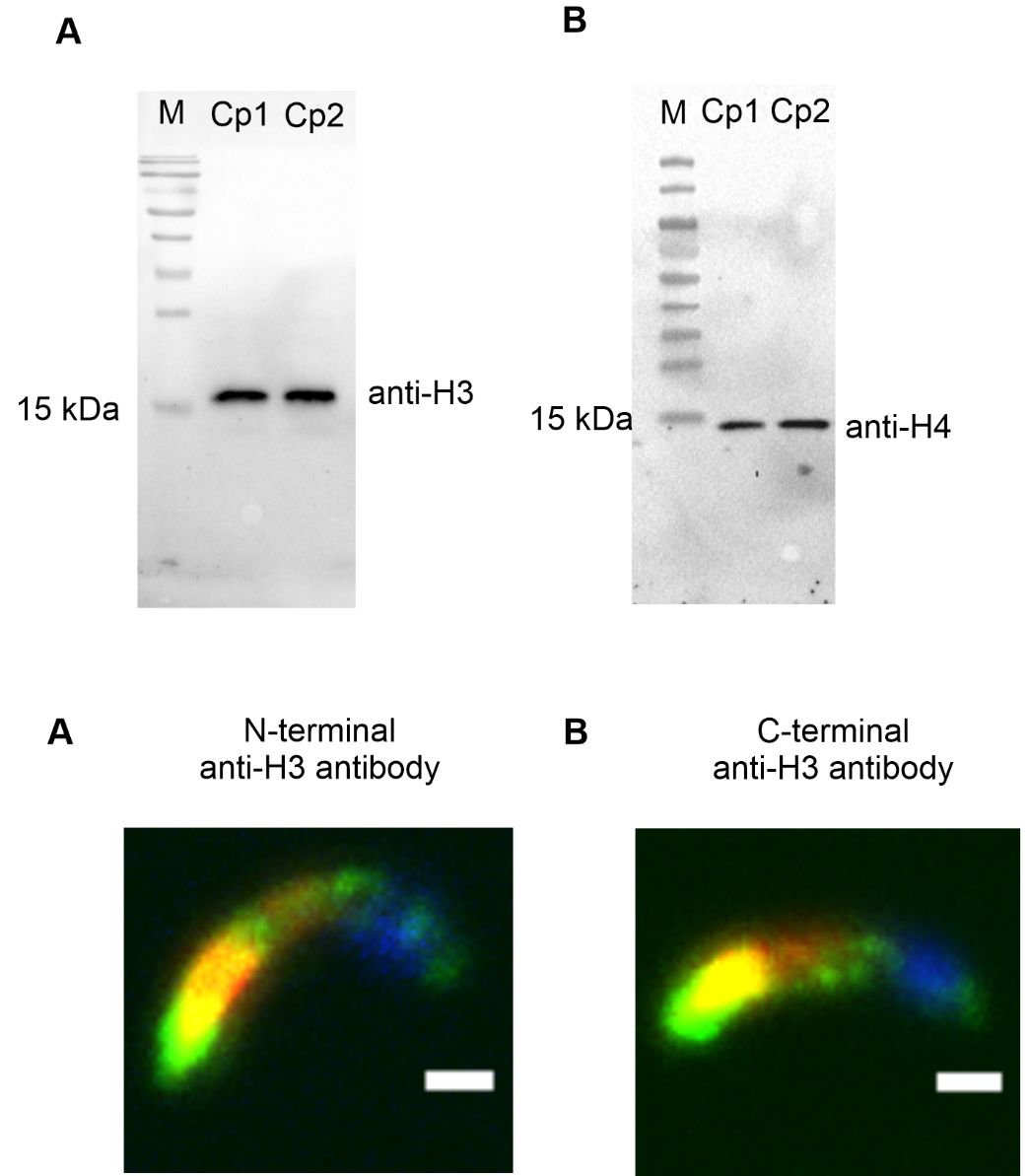


**Supplementary Figure 4**. Immunofluorescence analysis of histone 3 protein in *C. parvum* sporozoites. (A) Co-staining with anti-N terminal-histone 3 antibodies (Ab18521) and (B) anti-C-terminal-histone 3 antibody (Ab1791) (green), anti-*Cryptosporidium* antibody (red) and DAPI (blue). Scale bar 1 µm.


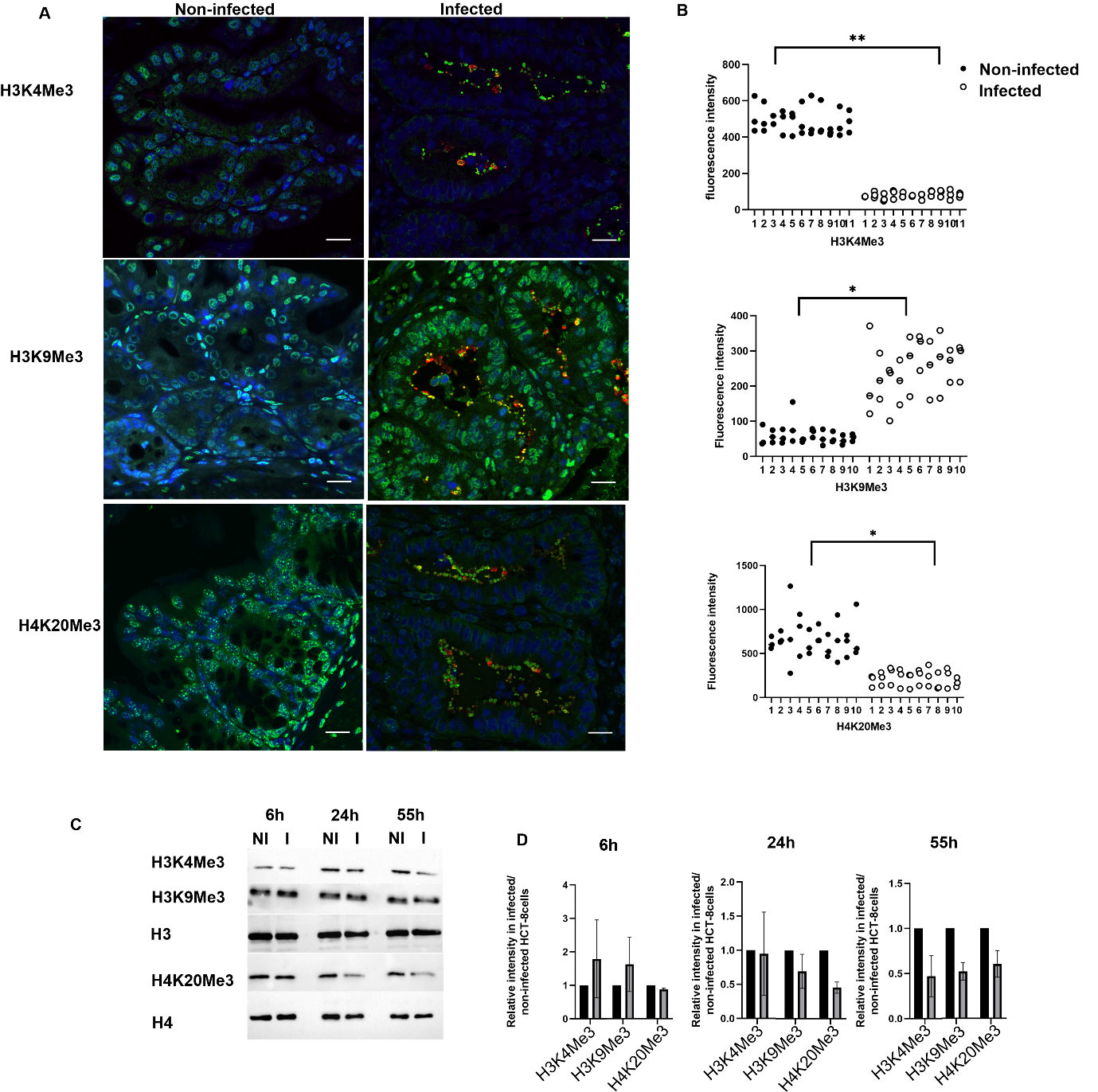


**Supplementary Figure 5.** Effect of *C. parvum* infection on methylation marks *in vivo* and *in vitro*. (A). Immunofluorescence analysis of histone methylation events during *C. parvum* infection *in vivo* at day 60 PI. Co-staining with anti- histone methylation antibodies such as H3K4Me3, H3K9Me3, H4K20Me3 (green), anti-*Cryptosporidium* antibody (red) and DAPI (blue) of the ileo-caecal region of *C. parvum* infected SCID mice. (B). Quantification of fluorescence intensity signals of anti-methylation antibodies in *C. parvum* infected vs non-infected ileo-caecal tissue. The signal intensities were measured by nuclei. Numbers on the Y axis indicate individual nucleus. The black and white circles indicate uninfected and infected nuclei respectively. For the statistical analysis, a mixed regression model was created considering fluorescence quantification as the main outcomes and sample identifier as random effect. The difference in the fluorescence intensity signals was statistically significant (p<0.05). (C). Western blotting analysis of histone methylation events during *C. parvum* infection *in vitro* at 6h PI (trophozoite stage) 24 h PI (asexual stage), 55 h PI (sexual stages) after purification of histones from host cell. (D). The histograms represent relative intensity signals measured in infected HCT-8 cells with respect to non-infected HCT-8 cells. Each sample was normalized to the H3 used as internal control. The graph represents mean of triplicate values. NI – Non-infected HCT8 cells. I – Infected HCT8 cells. Scale bar – 20 µm. Results are representative of three independent experiments.

| Antibodies | ICC/ IHC (dilutions) | Western blotting (dilutions) |
| --- | --- | --- |
| Anti-Histone H3 (tri methyl K4) antibody - ChIP Grade ab8580 | 1:1000 | 1:1000 |
| Anti-Histone H3 (tri methyl K36) antibody - ChIP Grade ab9050 | 1:500 | 1:1000 |
| Anti-Histone H3 (tri methyl K27) antibody - ChIP Grade [mAbcam 6002] | 1:500 | 1:1000 |
| Anti-Histone H3 (tri methyl K9) antibody - ChIP Grade [ab8898] | 1:500 | 1:500 |
| Anti-Histone H4 (tri methyl K20) antibody [EPR17001(2)] - ChIP Grade | 1:1000 | 1:1000 |
| Anti-Histone H3 antibody [ab18521] (N-terminal) | 1:100 | 1:1000 |
| Anti-Histone H3 antibody - Nuclear Marker and ChIP Grade [ab1791] (C-terminal) | 1:100 | 1:1000 |
| Goat Anti-Rabbit IgG H&L (Alexa Fluor® 488)(ab150077) | 1:1000 | NA |
| A600FLR-20X Sporo-Glo™ | 1:50 | NA |
| Goat Anti-Rabbit IgG H&L (HRP) (ab6721) |  | 1:1000 |

**Supplementary Table 1**. Primary and secondary antibodies dilutions

Abbreviations: IHC: immunohistochemistry; ICC: Immunocytochemistry; NA: Non applicable

**Supplementary Table 2.** Primers used for RT-qPCR analysis of putative KMTs

| **Gene ID** | **Primers** | **Tm** | **Sequences** | **Fragment**  **size (bp)** |
| --- | --- | --- | --- | --- |
| cgd1-2170 | F | 60.1 | 5’ gctgaagcagtatcccgttgca 3’ | 80 |
|  | R | 58.2 | 5’ tcgtctttcatacccagttcttgc 3’ |  |
| cgd4-370 | F | 58.4 | 5’ tgtgtaatcgccggatctc 3’ | 91 |
|  | R | 59.7 | 5’ gctctttggcctcgttaagc 3’ |  |
| cgd4-2090 | F | 59.9 | 5’ gccagggaatttgggtttaacg 3’ | 84 |
|  | R | 59.7 | 5’ ttcatcggttgcaatccctcc 3’ |  |
| cgd5-400 | F | 58.9 | 5’ gaaagatcctgcggagtatgc 3’ | 116 |
|  | R | 58.5 | 5’ tcttcgagtccgacgca 3’ |  |
| cgd5-2340 | F | 58.2 | 5’ tgctaacgatggaagcgca 3’ | 84 |
|  | R | 58.3 | 5’ gatctaccttctctttcgtcataccac 3’ |  |
| cgd6-1470 | F | 58.3 | 5’ gtagcttgcctagattggaaagca 3’ | 92 |
|  | R | 59.5 | 5’ tggaataagtcctgttcctagctc 3’ |  |
| cdg7-5090 | F | 58.6 | 5’ agtgaatccacgacaaaaagctcc 3’ | 80 |
|  | R | 58.4 | 5’ cccatccaagaatgcttgg 3’ |  |
| cgd8-2730 | F | 58.2 | 5’ tgacggtagaaagtgctagga 3’ | 116 |
|  | R | 58.8 | 5’ cttgcttgatgaggaatgagagc 3’ |  |
| 18S | F | 58.2 | 5’ tgccttgaatactccagcatgg 3’ | 103 |
|  | R | 59.6 | 5’ tacaaatgcccccaactgtcc 3’ |  |

**Supplementary Table 3.** MolProbity statistics for 3D models of CpSETs

| **3D model** | **Clashscore^a^** | | **Ramchandran favoured** | **MolProbity Score^b^** | |
| --- | --- | --- | --- | --- | --- |
| CpSET1 | 2.24 | 99^th^ percentile | 95.04% | 1.62 | 92^nd^ percentile |
| CpSET2 | 2.91 | 98^th^ percentile | 89.22% | 1.73 | 88^th^ percentile |
| CpSET8 | 4.61 | 96^th^ percentile | 94.52% | 1.57 | 93^rd^ percentile |

^a^ Percentile score established with N =1784, considering all resolutions

^b^ Percentile score established with N=27675, considering structures at all resolution ranges

**Supplementary Table 4.** Pairwise structure comparison between generated models and templates

| **Structure**  **Parameters** | **CpSET1 onto 5F6L** | **CpSET2 onto 6J9J** | **CpSET8 onto5TEG** |
| --- | --- | --- | --- |
| RMSD (Å)^a^ | 0.3 | 0.6 | 0.4 |
| Number of superimposed residues | 136 | 139 | 144 |
| Total number of residues | 143 | 169 | 148 |
| Dali z-score^b^ | 24.4 | 22.2 | 25.8 |
| Percentage identity^c^ | 52 % | 43 % | 44 % |

^a^ RMSD is the root-mean-square deviation computed over the Cα atoms of superimposed residues.

^b^ A DALI z-score greater than 3 is considered as reflecting similar structures. Higher z-scores indicate more similar structures.

^c^ Percentage identity is calculated between structurally superimposed residue
